# IFNγ Response to *Mycobacterium tuberculosis*, Risk of Infection and Disease in Household Contacts of Tuberculosis Patients in Colombia

**DOI:** 10.1371/journal.pone.0008257

**Published:** 2009-12-14

**Authors:** Helena del Corral, Sara C. París, Nancy D. Marín, Diana M. Marín, Lucelly López, Hanna M. Henao, Teresita Martínez, Liliana Villa, Luis F. Barrera, Blanca L. Ortiz, María E. Ramírez, Carlos J. Montes, María C. Oquendo, Lisandra M. Arango, Felipe Riaño, Carlos Aguirre, Alberto Bustamante, John T. Belisle, Karen Dobos, Gloria I. Mejía, Margarita R. Giraldo, Patrick J. Brennan, Jaime Robledo, María P. Arbeláez, Carlos A. Rojas, Luis F. García

**Affiliations:** 1 Grupo de Epidemiología, Universidad de Antioquia, Medellín, Colombia; 2 Escuela de Microbiología, Universidad de Antioquia, Medellín, Colombia; 3 Grupo de Inmunología Celular e Inmunogenética, Universidad de Antioquia, Medellín, Colombia; 4 Departamento de Pediatría, Universidad de Antioquia, Medellín, Colombia; 5 Centro Colombiano de Investigación en Tuberculosis, Medellín, Colombia; 6 Corporación para Investigaciones Biológicas, Medellín, Colombia; 7 Department of Microbiology, Immunology and Pathology, Colorado State University, Fort Collins, Colorado, United States of America; 8 Dirección Seccional de Salud de Antioquia, Medellín, Colombia; McGill University, Canada

## Abstract

**Objectives:**

Household contacts (HHCs) of pulmonary tuberculosis patients are at high risk of *Mycobacterium tuberculosis* infection and early disease development. Identification of individuals at risk of tuberculosis disease is a desirable goal for tuberculosis control. Interferon-gamma release assays (IGRAs) using specific *M. tuberculosis* antigens provide an alternative to tuberculin skin testing (TST) for infection detection. Additionally, the levels of IFNγ produced in response to these antigens may have prognostic value. We estimated the prevalence of *M. tuberculosis* infection by IGRA and TST in HHCs and their source population (SP), and assessed whether IFNγ levels in HHCs correlate with tuberculosis development.

**Methods:**

A cohort of 2060 HHCs was followed for 2–3 years after exposure to a tuberculosis case. Besides TST, IFNγ responses to mycobacterial antigens: CFP, CFP-10, HspX and Ag85A were assessed in 7-days whole blood cultures and compared to 766 individuals from the SP in Medellín, Colombia. Isoniazid prophylaxis was not offered to child contacts because Colombian tuberculosis regulations consider it only in children under 5 years, TST positive without BCG vaccination.

**Results:**

Using TST 65.9% of HHCs and 42.7% subjects from the SP were positive (OR 2.60, p<0.0001). IFNγ response to CFP-10, a biomarker of *M. tuberculosis* infection, tested positive in 66.3% HHCs and 24.3% from the SP (OR = 6.07, p<0.0001). Tuberculosis incidence rate was 7.0/1000 person years. Children <5 years accounted for 21.6% of incident cases. No significant difference was found between positive and negative IFNγ responders to CFP-10 (HR 1.82 95% CI 0.79–4.20 p = 0.16). However, a significant trend for tuberculosis development amongst high HHC IFNγ producers was observed (trend Log rank p = 0.007).

**Discussion:**

CFP-10-induced IFNγ production is useful to establish tuberculosis infection prevalence amongst HHC and identify those at highest risk of disease. The high tuberculosis incidence amongst children supports administration of chemoprohylaxis to child contacts regardless of BCG vaccination.

## Introduction

Tuberculosis (TB) is still a major cause of illness and death worldwide. In 2006, there were an estimated 9.2 million new cases of the disease and more than two billion people were expected to be infected with *Mycobacterium tuberculosis* [1]. Mathematical projections estimate that, even with sustained implementation of conventional control interventions, TB is not declining fast enough to achieve the goals proposed of halving the prevalence by 2015 and eliminating it by 2050 [2]. In addition to providing supervised therapy to cases, new tools to prevent infection and reduce transmission are required to accelerate progress in TB control [3]. In countries with medium to high TB prevalence, where a large percentage of cases are due to recent transmission, household contacts (HHCs) of pulmonary TB cases are at particularly high risk [4] and constitute an important target for early preventive alternatives in TB control [5]. Since the highest risk of disease development concentrates during the first two years after infection, identification of factors associated with infection and prognosis of disease development in recently exposed individuals is a highly instrumental and desirable approach for improving TB control [5].Traditional models of TB epidemiology based on longitudinal studies were performed several decades ago, using tuberculin skin testing (TST), indicate that 5–10% of recently infected contacts develop active disease within the subsequent 2–5 years after exposure to an infectious source, while another 5–10% percent develop TB some time during the rest of their lives [6]. However, there is a need of information coming from population based studies in disease endemic settings to revise such estimates under current epidemiological conditions.

Traditionally, *M. tuberculosis* infection levels have been estimated using TST; however, the purified protein derivative (PPD) is a mix of more than 200 proteins which presents cross reactivity with *M bovis* BCG and most environmental mycobacteria [7]. Comparative analysis of the mycobacterial genome allowed the identification of antigens coded by a region present in *M. tuberculosis* but absent in all BCG strains and all but four environmental mycobacteria [8], [9]. This region of differentiation, RD1, encodes CFP-10 and ESAT-6, which induce IFNγ production in mononuclear cells or whole blood cultures of infected individuals, thus IFNγ release assays (IGRAs) have been extensively used to diagnose latent TB infection (LTBI) [10]. Use of these highly specific assays in countries with high levels of endemicity, where BCG is still widely administered, would allow for a more refined understanding and identification of the factors associated with *M. tuberculosis* infection. Although the potential of RD1 antigens for establishing disease prognosis has been proposed [11] and some studies have begun to address it [12]–[16], there is still a lack of evidence from representative, population based studies.

In addition to RD1 antigens, several proteins involved in mycobacterial latency and immune reactivity have been characterized: HspX, also known as α-crystalline, is up-regulated during the bacterial stationary phase of growth, and is thought to be a key player in maintaining latency in the human host [17]. Antigen 85A (Ag85A) is a potent immunogen that has been proposed as a vaccine candidate [18].

The present study addresses whether IFNγ production in response to CFP-10, alone or in combination with HspX, Ag85A and CFP (a non-specific *M. tuberculosis* culture filtrate protein preparation) could identify HHCs at highest risk of developing disease within the first two years after exposure to an infectious source in Medellín, Colombia. Medellín's greater metropolitan area has a population of more than four million inhabitants [19] with a TB incidence of 27.7/100,000 (http://www.dssa.gov.co/htm/event_3.html). We present evidence that, as measured by IFNγ response to CFP-10, there is a high prevalence of infection in HHCs and that high HHC IFNγ producers in response to CFP-10 are at higher risk of developing TB than lower producers early after exposure to a pulmonary TB case.

## Methods

### Ethics Statement

All study's procedures and written consent forms were approved by the ethics review board of Universidad de Antioquia's Facultad de Medicina.

### Participants

**Figure 1:**
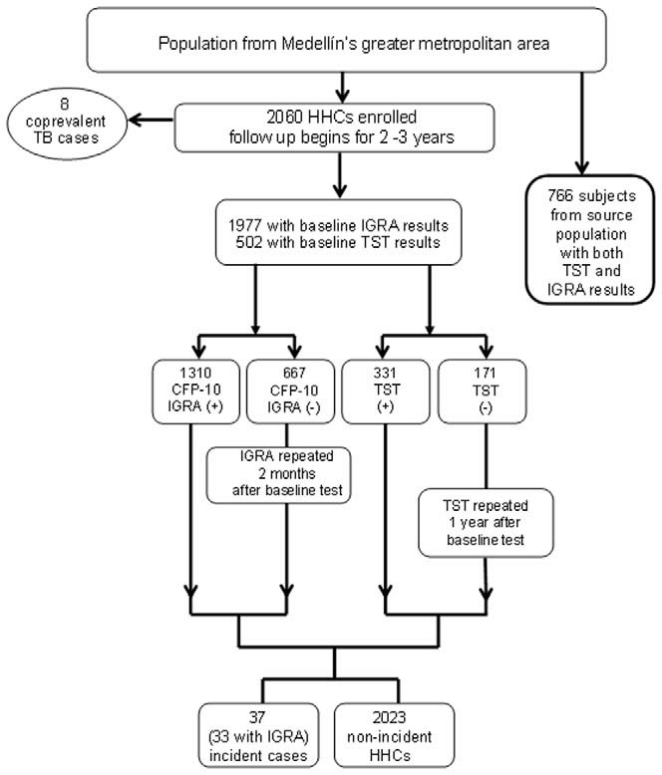
Study profile. HHCs = Household contacts, IGRA = IFNγ Release Assay, TST = Tuberculin Skin Test, SP = source population, CFP-10 = Culture Filtrate Protein-10.

A cohort of 2060 household contacts of smear positive pulmonary TB cases was assembled in Medellín's greater metropolitan area in Colombia, between March of 2005 and December 2006 (Figure 1). Sample size was estimated expecting 50% infection and 5% TB incidence amongst infected HHCs during the first 2 years after exposure. Under these conditions, a cohort of 2000 HHCs would allow for 82% power and 95% confidence to detect a difference of at least 0.80 in the TB incidence of the two groups of IFNγ producers (non-responders vs. responders) at baseline. Four hundred and thirty three (433) sputum smear positive pulmonary TB patients were recruited at the health centres where they were diagnosed. Mycobacterial culture was performed to 396/433 (91.5%) samples and 6.3% were culture negative. Only cases that were older than 15 years of age and had at least one HHC were considered as index cases and asked to participate. Bacteriological confirmation of TB was performed by detection of acid fast bacilli (AFB) at the local TB control program's laboratories and confirmed by the research team's Microbiology laboratory at Corporación para Investigaciones Biológicas (CIB) by sputum microscopy and culture. Patients were interviewed by a physician and an auxiliary nurse specifically trained for enrollment and follow-up of study subjects. Informed consent was obtained for participation in the study as index cases and to invite the patient's HHCs to enrol in the study. After written informed consent was provided, a structured interview was performed to ascertain the number of household contacts the patients had during the symptomatic period and first two weeks on TB medications, as well as exposure information related to the index case.

After approval by the index case, household contacts were visited and their eligibility was verified excluding those that had immediate plans to live outside the study area. After offering a thorough description of the study, a written informed consent was obtained from HHCs, and in minors, consent was granted by parents or responsible adult guardians. Demographic, social and exposure information was collected by administration of a structured questionnaire and physical household conditions such as number of rooms, total and index case's room area and volume, as well as number of doors, patios and windows in the household, were recorded. Socioeconomic strata (SES) defined by the local public services provider (Empresas Públicas de Medellín) were also registered. Time and magnitude of HHCs exposure to the index case was ascertained. A household contact was considered to be someone who had spent time regularly (weekly) in the same household as the index case for at least a month prior to the time when the index cases' diagnosis was confirmed. Inclusion criteria were: meeting the household contact definition, not having immediate plans to live outside Medellín's metropolitan area and provision of written informed consent. Exclusion criteria were: high level of suspicion of active TB or diagnostic work up for TB in progress.

Health status upon enrollment was established by physical examination performed by a physician and specific enquiry on immunosuppressive conditions related to medication intake and concurrent diseases. Contacts that were found to have symptoms suggestive of TB were studied to diagnose or rule out the presence of disease upon study entry. A co- prevalent case was defined as someone who was diagnosed with TB within two months after enrollment of the index case [14]. Blood samples were obtained from HHCs and processed as described below. Also, TST was performed in a consecutive sample of 502 HHCs after blood was obtained (see below). In addition to BCG scar ascertainment, mothers and adults responsible for all children in the study were asked to provide a copy of the child's vaccination certificate and inquired whether children had received vaccinations at birth.

Participants were followed every 3 months by phone, every 6 months by house visits and when they reported having TB compatible symptoms. In addition to extensive health education about TB, two telephone numbers were provided to study participants so they could report any signs or symptoms compatible with TB as soon as they became apparent. Whenever there was clinical suspicion of TB, particularly in TST positive children, all diagnostic procedures were oriented towards achievement of early disease detection. If diagnostic aids were not readily available through the TB Control Program, these were paid and provided by the study. HHCs that presented clinical symptoms and signs of pulmonary TB during follow-up were studied by chest X-rays and confirmed by AFB staining and culture from sputum samples in adults and gastric aspirate in children. Extrapulmonary and paediatric TB cases were diagnosed following American Thoracic Society's [20] and the Stop TB Partnership Childhood TB subgroup's guidelines [21], respectively. The definitive diagnosis of secondary TB cases in children was established by two experienced infectious disease paediatricians (CA, AB). All cases confirmed were referred to the local TB Control Program.

A cross-sectional survey was performed by random sampling of the source population (SP) in the areas which contributed most of the households enrolled in the HHC cohort. Four communities from Medellín, comprising the North-Eastern zone of the city, and two neighbouring towns (Bello and Itaguí) were sampled independently, stratifying by age and sex so as to reflect the distribution of these variables in the cohort's population. A probabilistic multistage sampling strategy was performed taking individual subjects as the sampling unit and the three stages being county, household, and individual. One person per household was selected on the basis of pre-established age and sex quotas. After obtaining written informed consent, each person was interviewed and a structured questionnaire was completed which contained demographic and TB risk related information. Ten millilitres of venous blood were also drawn and TST was performed on each study participant. Of 771 individuals sampled, 766 had complete information. These were estimated to allow for comparisons with 95% confidence level and 80% power.

### *Mycobacterium tuberculosis* Culture and Identification

*M. tuberculosis* cultures from index and incident active TB cases were derived from sputum samples in adults and from gastric fluid aspirate in children. Mycobacterial culture was performed in liquid medium (MGIT960 ® BD) and Lowenstein-Jensen (LJ) agar by standard procedures. Identification of mycobacteria species were performed using conventional phenotypic tests [22].

### Whole Blood Cultures

The method described by Dockrell *et al* [23], [24] was used for whole blood culture from all HHCs, SP subjects, all incident cases at the time of TB diagnosis and the 50 HHCs controls that did not develop active tuberculosis after two years of follow-up. In addition, HHCs who were non-responders at the first assessment were studied again 3–4 months later. Five to ten millilitres of venous blood were collected in vacuum-tainers containing sodium heparin. Whole blood was diluted 1∶10 in RPMI-1640 culture medium (Invitrogen, Grand Island, NY), supplemented with 100 U/ml of penicillin, 1 µg/ml of streptomycin and 2 mM L-glutamine. Diluted blood (200 µl/well) was dispensed in 96-well plates (Corning Costar Inc, Corning, NY, USA) and stimulated with 10 µg/ml of *M. tuberculosis* culture filtrate protein (CFP), 5 µg/ml of recombinant CFP-10, a *M. tuberculosis* specific RD1 antigen [8], [9], 5 µg/ml of recombinant Ag85A, a T-cell immunodominant antigen [18], and 10 µg/ml of recombinant alpha crystallin (HspX) a latency-associated antigen [17]. All antigens were produced and provided by Colorado State University's Mycobacteria Research Laboratory, Fort Collins, CO through the “Tuberculosis Vaccine Testing and Research Material Contract No. HHSN26266400091c. Non stimulated wells were used as controls. Cultures were incubated at 37°C and 5% CO_2_ for 7 days. At the end of the incubation period, supernatants were collected and stored at −70°C until used.

### Measurement of IFNγ by ELISA

IFNγ concentration in the culture supernatant of mycobacterial stimulated and non stimulated whole blood cultures were determined by ELISA using a commercial kit (Duo-set R&D Systems, Minneapolis, MN) following manufacturer's instructions. Readings were performed in an ELISA reader (ELx800NB, BioTek Instruments Inc, Winooski, VT) at 450nm and IFNγ concentration was established against a standard curve with known dilutions of the cytokine within a 15–1,000 pg/ml range. Supernatants exhibiting concentrations above the range included for the standard curve were diluted and quantified again.

### Tuberculin Skin Test

Two IU of RT23 tuberculin (Staten Serum Institute, Copenhagen, Denmark) in 100 µl were injected intradermally on the volar face of the left forearm. Transversal diameters of induration were measured 48–72 h later using the “ballpoint pen method” and following a pre-established standard operating procedure.

### Statistical Methods

Data was entered in a Microsoft Access database and statistical analysis was performed using SPSS version 17 (SPSS Inc. Chicago IL, USA), STATA 10.1 (StataCorp, College Station TX, USA) and Prism software version 5 (GraphPad San Diego CA, USA). Data entry, query generation and correction were performed on a daily basis. To establish the cut-off values for IFNγ production, the type of probability distribution that best fit the data from non stimulated cultures was identified as Gamma through visual inspection of Q-Q plots and Kolmogorov–Smirnov hypothesis testing [25]. Estimators for shape (α) and scale (β) parameters were estimated to be 0.025 and 0.042 respectively. The cut-off was established at 22 pg/mL after adjustment for within data correlations and allowing for an area under the curve of 99.9%. For estimation of geometric means, 1 was added to all IFNγ values. Net IFNγ production was calculated by subtracting values in non-stimulated cultures from stimulated ones.

Two different data sets were used for analysis of HHCs. The first included all HHCs with baseline assessments of IFNγ responses to mycobacterial antigens and the study of factors associated with IFNγ production in response to CFP-10, used as a surrogate biomarker of exposure to *M. tuberculosis* infection [26]. This set was used to compare HHCs to individuals from the source population. A second data set was used for analysis relating to disease development. The latter excluded co-prevalent cases and individuals without baseline IGRA results. After descriptive and bivariate analyses, a multivariate logistic regression model was constructed to assess factors associated with baseline *M. tuberculosis* infection levels in HHCs and SP. Adjustment for within household correlations was performed with Generalized Estimating Equations [27]. Categorical variable colinearity was assessed using Kendall's tau-B with α = 0.05. Odds ratios (ORs) were used to estimate the likelihood of infection in HHCs compared to that of individuals from the SP, using TST and IFNγ response to CFP-10 as surrogate markers of *M. tuberculosis* infection. The predictive potential of IFNγ response to mycobacterial antigens was first explored comparing the geometric means of IFNγ levels and the proportion of responders *versus* non-responders to each antigen in incident and non-incident HHCs. In addition, since it had recently been proposed by Andersen *et al* [11] that the risk of developing active TB varies according to the levels of IFNγ produced in response to RD1 antigens, we calculated this risk using the categories suggested by these authors, but adding a new one of non responders based on our cut-off. Thus, the following 4 categories were established: negative (<22 pg/mL), low (22–99 pg/mL), medium (100–999 pg/mL) and high (≥1000 pg/mL). Data analyses for assessment of disease prognosis were performed using Kaplan-Meier curves, hazard ratio (HR) and cumulative incidence rate estimations as well as Poisson multivariate regression (data not shown).

### Ethical Aspects

All study's procedures and written consent forms were approved by the Ethics Committee of Universidad de Antioquia's Facultad de Medicina; the study was also approved by the local health authorities (Dirección Seccional de Salud de Antioquia and the Secretaría de Salud de Medellín). At present the Colombian TB Control Program does not have a policy of offering Isoniazid Preventive Therapy (IPT), except in child contacts under 5 years of age, who are TST positive (≥10 mm) and have no evidence of BCG vaccination [28]. Given that several international guidelines clearly state the importance of offering IPT to young contacts [29]–[31], we inquired specifically about the possibility of offering IPT to child contacts and program officials reemphasized that Colombian regulations only allow IPT administration to TST positive children without BCG vaccination, Since in Colombia anti - tuberculous treatment can only be provided through the National Tuberculosis Control Program (NTP) we were unable to provide IPT to all child contacts.

It is worth noting that, in addition to regular follow up activities, monthly nutritional supplements were offered to all child contacts under 5 years old through the governmental MANA program (Mejoramiento Alimentario y Nutricional de Antioquia). This allowed us to monitor closely their health conditions through physical examinations and mother's enquiries. Health education activities, particularly regarding TB prevention and use of these supplements to improve nutrition, were also part of the activities provided every time children were evaluated and given supplements.

### Role of the Funding Sources

The study sponsors were not involved in study design, data collection, interpretation and analysis or in writing the manuscript. The corresponding author had full access to all data collected and had responsibility for the decision to submit the final draft for publication.

## Results

### Demographic and Clinical Characteristics of Index Cases, HHC and Source Population

Of 433 index cases recruited, 366 had HHCs that met inclusion criteria. Index cases were mostly (56.6%) men between 15 and 49 years of age (74.8%), predominantly belonging to the lowest 3 socioeconomic levels (63.4%) and 10.1% of them reported having had TB previously. Seventy two percent (72.2%) had BCG scars (Table 1). Their median time to diagnosis was 79 days (IQR: (43–156) and 62.3% had high AFB loads (++ or +++) (data not shown). Amongst 355 index cases tested, 14 (3.9%) had a positive HIV ELISA result and 14 of 289 (4.8%) had multidrug resistant TB.

**Table 1:** Characteristics of household contacts (HHCs) of pulmonary tuberculosis cases and individuals from their source population (SP).

Characteristic	Index Cases* (n = 366)	Household Contacts (n = 2060)	Source Population (n = 771)	p value†
**Sex (%)**				
Female	43.4	57.2	55.7	
Male	56.6	42.8	44.3	0.519
**Age (years)**				
**Median (IQR) (%)**	36 (24–50)	22 (10–42)	22 (11–46)	
≤4	0.0	11.5	11.1	
5–14	0.0	24.5	23.2	
15–24	25.1	18.6	18.1	
25–49	49.7	28.5	27.0	
50–64	16.9	10.3	15.5	
≥65	8.2	6.6	5.0	0.295
**Socioeconomic Stratum (%)** ‡				
−1	16.2	16.3	18.9	
−2	47.2	51.7	54.8	
−3	32.7	28.4	26.1	
−4	2.8	2.8	0.1	
−5	0.8	0.7	0.0	
−6	0.3	0.1	0.0	0,001
**BCG scar (%)**				
Yes	72.2	78.3	73.5	
No	27.8	21.7	26.5	0.008
**History of tuberculosis prior to study (%)**				
Yes	10.1	2.5	0.8	
No	89.9	97.5	99.2	0.006

*Only index cases that lead to household contacts included.

†p value (two tailed) refers to comparison of characteristics in HHCs and individuals from SP.

‡SES categories as defined in Methods.

The median number of persons per household was 5 (IQR 4–6). No significant differences in age and sex were found between HHCs and individuals from the SP (Table 1). Compared to subjects from the SP, a higher percentage of HHCs were vaccinated with BCG and had history of TB disease. Amongst those from the SP, 13.2% recalled having contact with someone with TB and 5.2% reported having lived with such person (data not shown).

Of 2060 contacts enrolled (Figure 1), 1977 had baseline IFNγ results and 502 also had TST. In the SP, 766/771 (99.3%) subjects had IFNγ and TST results in addition to complete sociodemographic data. Amongst the 2060 HHCs followed, after two years, 27 (1.3%) were lost to follow-up, 32 (1.6%) dropped out, 37 (1.8%) developed TB and 34 (1.7%) died from other causes.

### Tuberculin Skin Test Reactivity in HHC and Source Population

Since TST is the traditional method to measure mycobacterial infections, it was applied to a sample of HHCs and all individuals from the SP. Using a 10 mm threshold, 65.9% of HHCs were positive, compared to 42.7% subjects from the SP (OR 2.60, 95% CI 2.06–3.28, p<0.0001). Using 5 mm cut-off, 77.3% and 49.1% were TST positive, respectively (OR 3.53 95% CI 2.74–4.54, p<0.0001). Amongst 146 TST negative subjects at baseline who were retested after one year, 65 (44.5%) converted using 10 mm threshold and 43 (29.5%) converted according to the ATS/CDC criterion [32]. Median difference in induration diamaters between the first TST and the subsequent one after 1 year was 6 mm (Wilcoxon sign rank test p<0,001).

### IFNγ Production by HHC and Source Population in Response to Mycobacterial Antigens

**Figure 2:**
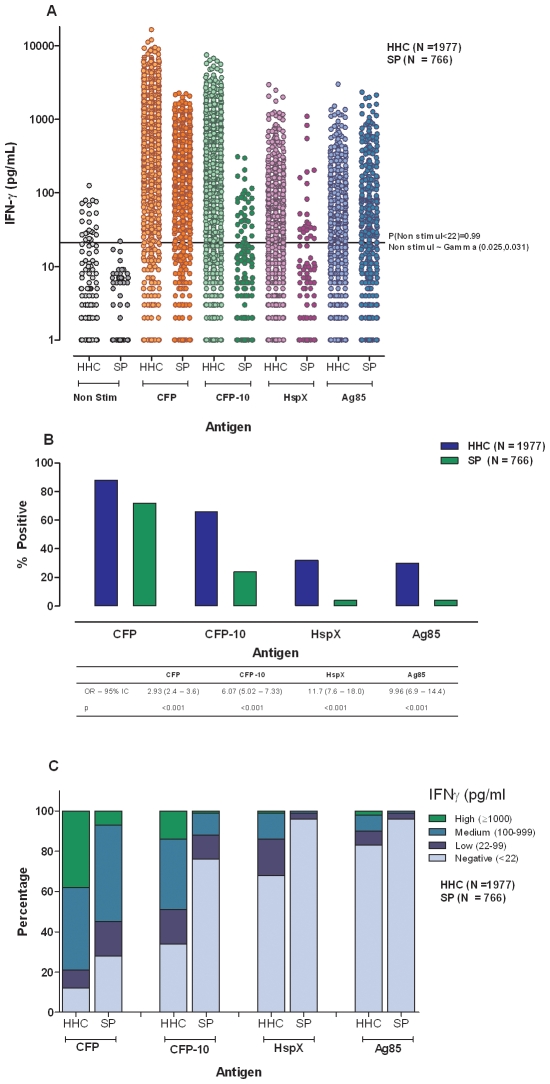
IFNγ production by HHCs and SP. IFNγ production in whole blood cultures stimulated with four mycobacterial antigens in household contacts and source population. A. IFNγ levels produced in non stimulated and CFP, CFP-10, HspX and Ag85A, stimulated cultures. B. Bar graphs depicting the percentage of positive responders and OR in HHCs and SP. C. Stacked bars represent a modification of the IFNγ production levels proposed by Andersen *et al* [11] showing: Negative: <22pg/mL, Low: 22–99 pg/mL Medium: 100–999 pg/mL, High: ≥1000 pg/mL. HHCs: Household Contacts; SP: Source Population.

In the last decade IFNγ production in response to specific mycobacterial antigens have received much attention as a more reliable and specific biomarker of *M. tuberculosis* infection [26], [33]. Thus we compared IFNγ production in the two populations in response to specific CFP-10 antigen [34] as well as to non-specific Ag85A [35], latency associated HspX [17] and the mixture of culture filtrate proteins (CFP) as an *in vitro* equivalent to TST. Geometric means of IFNγ production levels were significantly (p<0.0001) higher in HHCs than in the SP in response to CFP (495.9 *versus* 154.7 pg/mL), CFP-10 (146.3 *versus* 44.4 pg/mL), HspX (31.9 *versus* 12.7 pg/mL), and Ag85A (31.5 *versus* 11.9 pg/mL) (Figure 2A). With all antigens assayed, the likelihood of having a positive IFNγ response was higher (p<0.001) in HHCs than in the SP subjects. With CFP, there were 88.3% positive HHCs and 72.0% in the SP (OR = 2.93 95% CI 2.4–3.6). In response to CFP-10, 66.3% of HHCs were positive in contrast to 24.5% of the subjects from the SP (OR = 6.07 95% CI 5.02–7.33). IFNγ responses to HspX and Ag85A were 32.0% and 30.2% in HHCs while only 3.9% and 4.2% in the SP (OR = 11.7 95% CI 7.6–18.0 and OR = 9.96 95% CI 6.9–14.4), respectively (Figure 2B). As described above, a modification of the IFNγ level categorization proposed by Andersen *et al* [11] was used to further analyse the magnitude of IFNγ responses and the most striking difference between the two populations was found in CFP-10 responses, while 49.2% of HHCs presented medium to high levels of IFNγ production, only 12.6% of SP subjects exhibited those two levels (trend χ^2^; p<0.0001) (Figure 2C).

Of note, amongst HHCs who were initially negative for IFNγ production in response to CFP-10, 43.1% became positive when retested, 42.0% of them with increases ≥30% of initial values [36]. Thus, taking into account both measurements, HHCs positivities were 90.1%, 79.4%, 37.8% and 31.6% for CFP, CFP-10, HspX and Ag85A, respectively.

### Effect of Age on IFNγ Production and Tuberculin Skin Test Reactivity

**Figure 3:**
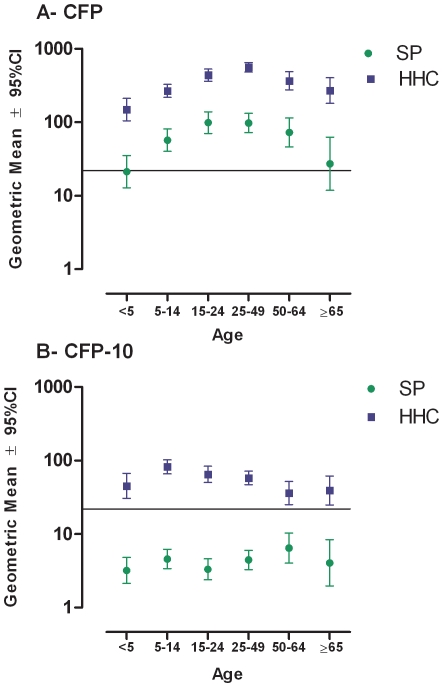
IFNγ production levels by age. Geometric means of IFNγ production in response to CFP (A) and CFP-10 (B) by age in household contacts (HHCs) and individuals from source population (SP). Horizontal lines depict the cut-off value (22 pg/mL).

Considering the wide range of age amongst the HHCs and that responses in young HHCs maybe more conditioned by the recent exposure to index cases, than in adults in whom past exposure to other sources cannot be ruled out, it was important to determine whether IFNγ production and TST varied with age, as previously reported [37]. Geometric means of IFNγ responses to CFP and CFP-10 were higher in HHCs than the SP in all age groups (Figure 3). A tendency of mean IFNγ responses to CFP to peak in young adults between 25 and 49 years and decline after 50 years of age was observed in the two populations studied (Figure 3A). The same tendency was not observed with CFP-10 in either HHCs or the SP (Figure 3B). In the SP, IFNγ responses to CFP-10 in all age groups were below the 22pg/mL cut-off.

Correlation of IFNγ responses to CFP and CFP-10 (pg/mL) *versus* TST (mm induration) were significant in HHCs and the SP using both antigens (p<0.0001). Overall agreement was higher in HHCs than in the SP with both antigens. Interestingly, a tendency of correlation coefficients to decrease with increasing age until 50 or 65 years was observed with both antigens in HHCs (Table 2).

**Table 2:** Correlation between TST and IFNγ production in response to CFP and CFP-10 by age in household contacts (HHCs) and individuals from source population (SP).

Age	HHCs (n = 502)		SP (n = 766)	
	CFP	CFP-10	CFP	CFP-10
≤4	0.78*	0.74	0.42	0.45
5–14	0.69	0.64	0.48	0.29
15–24	0.44	0.48	0.36	0.26
25–49	0.39	0.42	0.44	0.31
50–64	0.73	0.38	0.57	0.29
≥65	0.57	0.45	0.54	0.55
Total	0.58	0.51	0.49	0.30

*Spearman correlation coefficient *r*. All p values<0.001.

†TST (mm of induration); IFNγ (pg/mL).

### Effect of Exposure to the Index Case on IFNγ Production

To assess whether the CFP-10 induced IFNγ response was conditioned by exposure to the index case, positive and negative responders were compared regarding several markers of exposure (Table 3). HHCs that had positive baseline IFNγ in response to CFP-10 were more likely than those with negative responses to have slept in the same room as the index case while symptomatic without treatment (OR 1.26 p = 0.043), to live in households with less than 12m^2^ per person (OR 1.47 p = 0.002), and with less air volume per person (p = 0.008 χ^2^ for trend).

**Table 3:** Characteristics associated with baseline levels of IFNγ production in response to CFP-10 in HHCs.

Variable	OR_GEE_ *	95% CI	p
**Sex**			
Male	1.30	1.08–1.56	
**Age (years)**			
≤4†	1.00		
5–14	1.89	1.36–2.61	0.0001
15–24	1.68	1.19–2.36	0.003
25–49	1.62	1.18–2.22	0.003
50–64	1.19	0.82–1.75	0.364
≥65	1.33	0.85–2.08	0.206
**Slept in same room as case**	1.26	1.01–1.57	0.043
**Crowding** ‡ **(3 or more persons per room)**	1.17	0.87–1.58	0.295
**Crowding** ‡ **(less than 12m^2^ per persons)**	1.47	1.15–1.89	0.002
**Crowding gradient (m^2^/room)** §			
Greater than 30†	1.00		
21.9–30.0	0.74	0.52–1.06	0.106
16.1–21.8	1.08	0.74–1.56	0.699
16.0 or less	1.04	0.72–1.50	0.838
**Crowding (volume per person – m^3^)** §			
50.4 or more†	1.00		
29.4–50.3	0.91	0.63–1.31	0.607
19–29.3	1.18	0.82–1.71	0.368
18.9 or less	1.30	0.90–1.87	0.160

*Crude Odds Ratios adjusted for intra-household correlations with Generalized Estimating Equations (assumptions: exchangeable matrix, logit link function and binomial family).

†Reference category.

‡According to definition identified in reference [53].

§Categories are quartiles of the air volume distribution.

### Incidence of Active TB amongst HHC

The overall incidence rate of tuberculosis development in HHCs was 7.0/1000 person years. The incidence proportion was highest in children under 5 years of age (Table 4). Twelve of thirty seven (32.4%) incident cases were under 15 years of age and 8/37 (21.6%) under five. Most incident cases (75%) occurred after six months of follow-up, 40.5% of them shared bedroom with the index case and only 67.6% had a BCG scar compared to 78.3% in the whole cohort. Comparison of geometric means of IFNγ produced by incident cases and non-incident HHCs only showed a significant difference with HspX: 19.2 pg/mL (95% CI 8.3–44.9) in incident cases *versus* 35.6 pg/mL (95% CI 32.3–39.1) in non-incidents (p = 0.009) (Table 5).

**Table 4:** Incidence of active tuberculosis in household contacts according to age.

Age	N	Cases	Percentage of TB cases	Incidence proportion	Person-years	Incidence rate (#/1000 person-years)
**≤4**	237	8	21.6	3.4	619.9	12.9
**5–14**	505	4	10.8	0.8	1342.0	3.0
**15–24**	381	7	18.9	1.8	985.5	7.1
**25–49**	585	7	18.9	1.2	1498.0	4.5
**50–64**	209	7	18.9	3.3	510.8	13.1
**≥65**	135	4	10.8	3.0	315.3	10.6
**Total**	2052	37	100.0	1.8	5272.3	7.0

**Table 5:** Comparison of baseline IFNγ production by TB incident and non–incident HHCs in response to the antigens used.

Antigen	Geometric Mean (CI 95%)	Geometric Mean (CI 95%)		% (n/n)	% (n/n)	χ^2^ †
	Incident	Non-incident	p value*	Incident	Non-incident	p value
**CFP**	562.7 (324.7–975.1)	554.5 (515.3–596.7)	0.958	87.9 (29/33)	88.3 (1704/1929)	0.935
**CFP-10**	248.9 (133.9–462.6)	188.8(174.4–204.4)	0.278	78.8 (26/33)	66.0 (1281/1940)	0.124
**HspX**	19.2 (8.3–44.9)	35.6 (32.3–39.1)	0.009	33.3 (11/33)	32.0 (611/1912)	0.866
**Ag85A**	29.2 (12.5–68.0)	34.5 (31.0–38.3)	0.549	39.4 (13/33)	30.1 (583/1940)	0.246

*Student t - test.

†Chi-square (χ^2^) test.

### Predictive Value of IFNγ Production on the Development of Active TB in HHC

**Figure 4:**
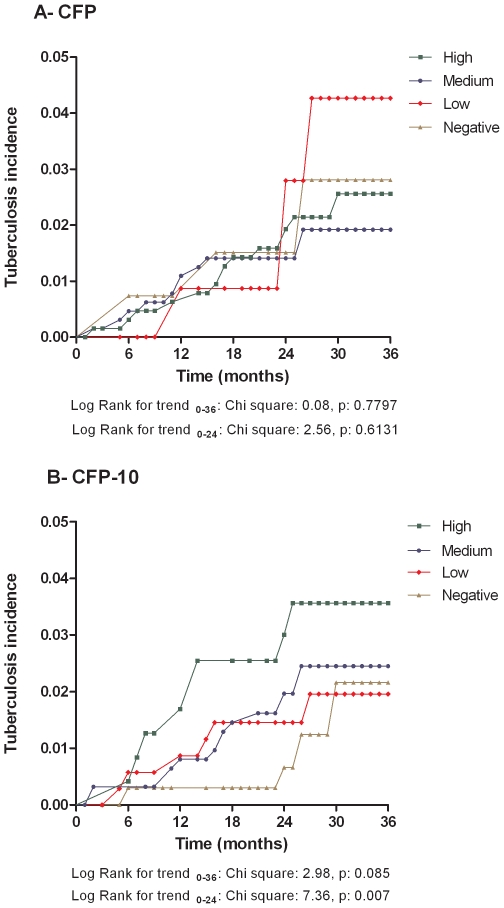
Hazard of TB development according to IFNγ production. Hazard levels of tuberculosis development taking the modified version of IFNγ production categories proposed by Andersen et al [11] as baseline predictors of disease. Negative: <22pg/mL, Low: 22–99 pg/mL Medium: 100–999 pg/mL, High: ≥1000 pg/mL. HHCs: Household Contacts SP: Source Population.

To explore whether IFNγ production in response specific (CFP-10) and non-specific (CFP) *M. tuberculosis* antigens at baseline had a predictive value, the risk of TB during the first two years of follow-up was assessed according to IFNγ responses as dichotomous and ordinal outcomes. TB incidence was higher, but not significant difference was found in positive IFNγ responders to CFP-10 in contrast to negative ones (HR 1.82 95% CI 0.79–4.20 p = 0.16). Using our modification of the IFNγ production categories proposed by Andersen *et al* [11], almost a 3-fold difference was found between the highest IFNγ producers at baseline compared with negative responders. Intermediate incidence rates were found in low and medium categories (Table 6). Further support for this finding was provided by the significant tendency of increasing hazard rates of disease development with increasing IFNγ production levels in response to CFP-10 observed during follow-up (Log Rank for trend p = 0.007) (Figure 4B). Disease incidence was found to be highest in strong responders to CFP-10, whilst those with medium and low levels of IFNγ production had intermediate incidence levels and the lowest incidence was found in non responders. Response to CFP was not associated with risk of developing disease (Figure 4A). Interestingly, within the first two years after exposure, HHCs with negative IFNγ responses to CFP-10 had low hazard level of disease development and their risk was distinctly different from the rest. However, from that time point on, hazard in the lowest responder group rose and at 3 years became very similar to those in the low and medium response categories (Figure 4B).

**Table 6:** Incidence of active tuberculosis in household contacts and IFNγ production levels at baseline.

IFNγ production*	N	Cases	Percentage of TB cases	Incidence Proportion	Person-years	Incidence Rate (#/1000 person-years)
Negative	666	7	21.2	1.05	1686.9	4.1
Low	338	6	18.2	1.78	857.9	7.0
Medium	688	12	36.4	1.74	1798.1	6.7
High	281	8	24.2	2.85	703.5	11.4
**Total Positive** †	1307	26	78.8	1.99	3359.6	7.7

*Negative: <22pg/mL, Low: 22–99 pg/mL Medium: 100–999 pg/mL, High: ≥1000 pg/mL.

†Total positive: (Negative + Low + Medium + High).

## Discussion

Herein we present evidence that levels of IFNγ response to CFP-10 assessed shortly after exposure to an infectious source may be predictive of tuberculosis development in a population of HHCs at high risk of infection. To our knowledge, this is the first population based study to uphold the model posed by Andersen *et al* [11]. These authors proposed specific categories of IFNγ levels produced in response to RD1 antigens that identify subgroups at differential risk of disease development. Several studies have inquired about the role of IFNγ responses to RD1 antigens for determining the prevalence of *M. tuberculosis* infection [38]–[40] and predicting disease development [13], [15], [16] in high risk populations; however, data coming from population based studies with sufficient power to assess disease prognosis has been limited [15], [16], [38]. Albeit our study was performed in a country with an intermediate level of TB incidence, we consider that the immunologic profiles associated with high levels of infection and disease development in HHCs maybe partly explained by a high intensity of exposure in this population.

Our findings are based on a cohort of HHCs with few losses and, compared to a representative sample of the SP, both with sizes large enough to provide an adequate degree of estimate precision and power. Besides the traditional TST, we used a novel, though extensively evaluated, biomarker of *M. tuberculosis* infection: IFNγ response to RD1 antigen CFP-10 and to other mycobacterial antigens, particularly Ag85A, HspX and CFP. TST results with two different cut-offs showed 2.6–3.5 fold differences between the two populations and IFNγ production in response to all antigens assessed was significantly higher in HHCs than in SP. The observation of similar estimates of *M. tuberculosis* infection when TST and IGRA for CFP-10 are used in HHCs but lower positivity with IFNγ response to CFP-10 in SP support that IFNγ measurement has higher specificity than TST, as has been extensively reported [12], [39], [41]–[45]. The consistently positive association of exposure related variables with positive IFNγ responses to CFP-10 indicates that *M. tuberculosis* may be responsible for such responses. In addition, the predominantly medium and high IFNγ production levels in response to CFP-10 found in HHCs, in contrast to mostly low and negative responses in the SP strongly suggest that mycobacterial stimulation in HHCs is predominantly driven by *M. tuberculosis*.

Exploration of IFNγ variations by age revealed a trend to increase responses up to adulthood with CFP, but not with CFP-10 in both HHCs and SP. This finding suggests that cumulative exposure to environmental mycobacteria and *M. bovis* BCG vaccination, besides *M. tuberculosis*, plays a role in determining responses to CFP, but not to the *M. tuberculosis* specific antigen CFP-10. In fact, IFNγ responses to CFP-10 in HHCs were above the cut-off point in subjects of all ages, but always below it in the SP. This differential pattern shows the ability of CFP-10 responses to discriminate between the risk of exposure to *M. tuberculosis* in HHCs and the SP, irrespective of age. The observation that IFNγ responses to CFP-10 by age failed to show a trend and that children exhibit IFNγ responses as high as those in young and old adults, confirms previous observations in Ugandan HHCs [45].

Additionally, in HHCs, correlations of IFNγ levels in response to CFP-10 and CFP, and TST indurations by age were highest in children in whom recent exposure to the index case is predominant. In contrast, in the SP the best correlations were found in the older age groups in whom cumulative exposure to *M. tuberculosis*, or other mycobacteria in the case of CFP induced responses, is likely to be highest. This provides further evidence of the *M. tuberculosis* specific nature of the responses to CFP-10.

An important difference between our study and others that have been performed using IFNγ responses is that the in-house assay performed by us incorporates a 7 day whole blood culture in contrast to the 24h incubation commonly used by commercial IGRAs [46], [47]. By virtue of the extended lymphocyte culture times used herein, these measures may primarily detect central memory responses while short incubation IGRAs commonly used, detect mostly effector memory T cell responses of recently activated lymphocytes [48], [49] [Marín *et al.* manuscript in preparation]. It has been argued that, in settings of high endemicity where a mixture of recent and old infections are commonly found, long term assays are more sensitive than those with shorter culture times [48]. Thus the incubation time used in our study may contribute to account for the high levels of IFNγ responses to CFP-10 observed in HHCs and the establishment of latent tuberculosis infection amongst HHCs.

The main goal of our study was to establish the value of IFNγ responses to CFP-10 as prognostic marker of tuberculosis disease development. Except for HspX-induced responses, univariate and bivariate analyses of IFNγ baseline levels did not show significant differences between incident and non-incident HHCs (Table 5). However, during the course of this study, Andersen *et al* [11] proposed a model in which IFNγ responses to RD1 antigens can be categorized in low, medium and high, where higher producers are at increased risk of disease development. Thus, we modified such categories adding a fourth level of negative responders (≤22 pg/mL) based on the cut-off value taking into account the gamma distribution found in the non-stimulated cultures. Strikingly, we found a significant trend to increase incidence of tuberculosis with increasing levels of CFP-10-induced IFNγ, but not with CFP. The marked differences in risk of disease progression in the negative responder group compared to the rest during the first 2 years, suggest that indeed these HHCs were not infected and shows the added value provided by this categorization. Interestingly, after 2 years post-exposure, the cumulative incidence in non-responders levelled out with those of low and medium IFNγ producers. The reasons why risk levels become similar after the first 24 months is unclear. We explored differences among subjects in the negative response group regarding socioeconomic, demographic and epidemiologically relevant variables but significant differences were not found (data not shown). Possible explanations for levelling out of risks of disease development in subjects with different levels of IFNγ production in response to CFP-10 could be related to *M. tuberculosis* re-exposures and slow responses due to co morbidities.

The more detailed assessment of the evolution of IFNγ levels produced by incident cases and non-incident HHCs in response to CFP and CFP-10 is in agreement with the model proposed by Andersen *et al* [11]. These authors proposed three stages in tuberculosis immunopathogenesis following *M. tuberculosis* infection: an initial phase of incipient disease characterized by a small peak of IFNγ production in response to RD1 antigens, which in those that initially control the bacillus, is followed by a decline and subsequent plateau of IFNγ levels throughout the duration of bacterial latency, only to rise again and reach the highest levels when active disease ensues. A few studies support this issue [50], [51]. Although our data provide support for the concept that high levels of IFNγ in response to RD1 antigens after exposure are indicative of higher risk of disease development during the first 2 years post exposure, the observation that most incident cases occurred 6–24 months after the diagnosis of the index case is not fully in line with the reasoning [11] which proposes that this model holds only for short term progressions to overt disease. In the case of child HHCs the exposure to the index case most probably resulted in primary infection and in a high proportion of them in post-primary disease. The high levels of conversions by TST and IGRA, as well as the high disease incidence rate ratios observed in children, suggest that a high proportion of disease may be due to recent transmission. In adult HHCs such exposure may result in reinfection, making it likely that a large proportion of our incident adult cases were due to reinfections rather than to reactivations.

Besides exploring responses to CFP-10 and CFP, we also assessed responses to antigens HspX and Ag85A. We found the greatest difference in IFNγ responses between HHCs and SP was marked by HspX and followed closely by Ag85A. The differences in responses to HspX can be explained by its known role in maintaining latency, but the reasons for the large differences in responses to Ag85A remain unclear to us. It must be noted that for stimulation in whole blood cultures we used recombinant proteins rather than peptides; however, Arend *et al* compared the response to the two type of CFP-10 preparations and found them to be equivalent [52]. In addition, though epitope mapping has been performed for these antigens, it is possible that other epitopes are present in the proteins. In whole blood cultures the use of proteins does not require the differentiation of MHC-I vs. MHC-II epitopes, which determines the response to particular peptides. Furthermore, in an *in vitro* T cell stimulation assay such as the one performed in this cohort, whole proteins typically give a more robust response than peptides (John T. Belisle personal communication).

Given the very high rates of tuberculosis development observed in children and the fact that we could not offer them IPT, as recommended by several international guidelines [29]–[31], the ethical implications of this study is a highly relevant issue. However, according to the Colombian TB Control Program [28] IPT can only be provided to children under 5 years of age with ≥10 mm induration in the TST test, no BCG vaccination, conditions that were not met by any of this cohort's children. In addition, the administration of all anti-TB treatments is restricted to the government operated TB Control program, a reason why our research team was unable to provide it.

We consider that, in spite of not being able to provide prophylaxis to children less than 5 years, our results serve to increase the visibility of this vulnerable population subgroup. Twenty two percent of incident cases in our study were children ≤4 years compared to only 3% of this age group detected through regular surveillance activities in Medellín (http://www.dssa.gov.co/htm/inciden.htm). Even though we did not offer IPT, we closely followed the cohort members resulting in a reduction of the median diagnostic delay from 2.6 months in index cases to 1.0 month in incident cases (data not shown). In addition, the results clearly demonstrate the high risk of infection and disease development in child HHCs. We consider that this finding underscores the need to focus on this population subgroup and strengthen contact tracing activities. Since this message was delivered by members of our group to health authorities in several occasions, Colombian health authorities decided to change the national health regulations to allow the provision of tuberculosis IPT to all child contacts less than 5 years of age regardless of vaccination status (statement issued by the Public Health Director of the Colombian Ministry of Social Protection).

Taken together our results clearly demonstrate that HHCs are a population at high risk of infection and disease development and show a marked difference in the risk of tuberculosis development depending on the levels of IFNγ production in response to CFP-10 shortly after exposure to *M. tuberculosis*. Since the identification of close contacts at highest risk of disease development is a highly desirable tool for more efficient tuberculosis control, the results presented herein constitute a remarkable step forward in the search for a biomarker of disease prognosis and open up the possibility of providing targeted IPT administration to child contacts based on IFNγ production in response to CFP-10 antigen.
